# Removal of hydrogen sulfide from biogas using activated carbon synthesized from different locally available biomass wastes - a case study from Palestine

**DOI:** 10.1080/21655979.2020.1768736

**Published:** 2020-05-28

**Authors:** Hassan Sawalha, Maher Maghalseh, Janna Qutaina, Kholoud Junaidi, Eldon R. Rene

**Affiliations:** a Renewable Energy and Environment Research Unit, Mechanical Engineering Department, Palestine Polytechnic University, Hebron, Palestine; b Renewable Energy and Environment Research Unit, Electrical Engineering Department, Palestine Polytechnic University, Hebron, Palestine; c Department of Environmental Engineering and Water Technology, IHE Delft Institute for Water Education, Delft, The Netherlands

**Keywords:** Hydrogen sulfide, packed-bed, adsorption, activated carbon, removal efficiency

## Abstract

The main aim of this study was to remove hydrogen sulfide (H_2_S) from biogas by adsorption using synthesized activated carbon prepared using locally available biomass. The effect of the type of precursors, impregnation reagent and bed height was studied in continuous reactors. Three types of biomass wastes (almond shells, eucalyptus and coffee grains) were collected, grinded, sieved, pyrolyzed at 500°C and impregnated with chemical reagents such as potassium hydroxide or zinc chloride. Adsorption tests were performed using a fixed bed filter filled with the produced activated carbon. The highest biochar yield of 36% was obtained eucalyptus followed by almond shells (28.5%) and coffee grains (24%), respectively. The highest adsorption capacity and removal efficiency were obtained with eucalyptus followed by almond shells and coffee grains, respectively. For instance, eucalyptus showed an adsorption capacity of ~690 (mg hydrogen sulfide/g adsorbent) followed by almond (230 mg hydrogen sulfide/g adsorbent) and coffee grains (22 mg hydrogen sulfide/g adsorbent). As an impregnation reagent, potassium hydroxide gave the highest adsorption efficiency and capacity than zinc chloride. Furthermore, the breakthrough time with KOH (180 min) was higher than ZnCl_2_ (70 min). Increasing the bed height during continuous breakthrough tests increased the adsorption capacity and hydrogen sulfide removal efficiency. The results of this study showed that the adsorption efficiency of the synthesized activated carbon and consequently the hydrogen sulfide removal efficiency could be fine-tuned by selecting an appropriate biomass precursor and proper impregnation reagent.

## Graphical abstract

**Figure:**
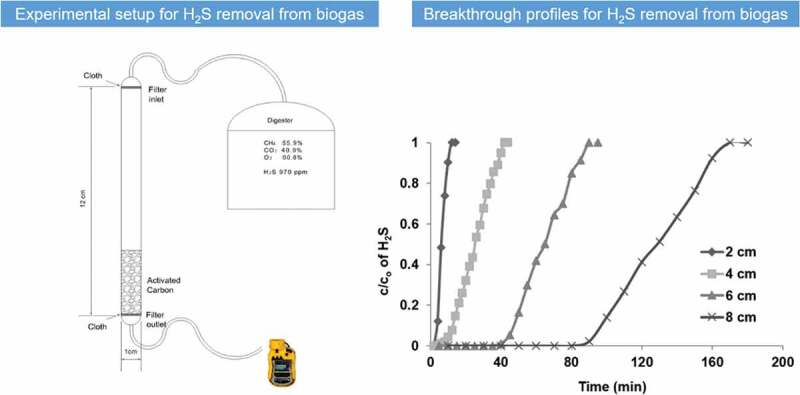

## 1. Introduction

Biogas is a promising renewable energy source that can replace the conventionally used nonrenewable fossil fuels in most of the developing countries (e.g. Palestine and India). Conventionally, biogas is commonly produced through the anaerobic digestion of different biomass feedstocks including animal, agricultural and organic food waste resides [1–5]. The produced biogas is mainly composed of methane, carbon dioxide and fractions of water vapor and hydrogen sulfide (H_2_S), i.e. H_2_S is an impurity of biogas [6]. The presence of H_2_S adversely affects the quality of the biogas and it has major environmental and technical drawbacks. For instance, at concentrations of 1000 to 2000 ppm, H_2_S can cause a collapse, coma and death from respiratory failure within a few seconds after one or two inhalations [7]. At concentrations of 100 to 200 ppm, H_2_S may cause a blurred vision and death following exposure for 1–8 h [7]. Moreover, dissolved H_2_S with concentration up to 50 ppm causes fermentation inhibition since it is toxic to the bacteria in the slurry of bioreactors [8]. Technically, H_2_S is corrosive and can significantly damage the metallic parts of the biogas facilities and electrical generator [9]. Furthermore, the electrical generation efficiency dramatically decreases when the H_2_S concentration in the biogas is very high. From an environmental perspective, the combustion of H_2_S during electrical generation forms sulfur dioxide (SO_2_), leading to the formation of a highly corrosive gas and it contributes as a major source of acid rains [9]. Therefore, the removal of H_2_S form the biogas is of high technical, environmental and health importance.

In the literatures, various biological, chemical and physical techniques have been successfully applied for the purification of biogas from H_2_S such as biological desulfurization, chemical absorption, water scrubbing, membranes and adsorption [8–14]. Adsorption is one of the most effective technologies applied in removing H_2_S from biogas streams. The high H_2_S removal efficiency achieved by adsorption makes it superior over other purification techniques. Besides, it is a rather inexpensive technique with wide range of choices of low-cost adsorbents.

During the adsorption process, the biogas is contacted with an adsorbent in which the H_2_S is collected on the surface and interior microstructure of the adsorbent. Several types of adsorbents have been proposed in the literature, including metal oxides, silica gel, zeolites, synthetic resins, and activated carbon [8,15–21]. Activated carbon has been widely used for the treatment of wastewater and waste gases. The high porosity and surface area of activated carbon provide a high capacity for the separation of pollutants from the liquids and gases present in the polluted environment. The activated carbon is commonly produced through the pyrolysis and impregnation of various biomass feedstock [22]. During pyrolysis, the biomass is thermally decomposed in the absence of oxygen at a controlled temperature to produce biochar [23]. The biochar, also called ‘charcoal’ in the literature, is the solid product from pyrolysis, or it is the carbonaceous residue after all the volatile matter leaves the biomass as gases and tar. The pyrolysis process is usually followed by an impregnation step. There are three types of activation: physical, chemical and physico-chemical activation [24].

Chemical impregnation is largely implemented in practice due to the lower impregnation temperatures and shorter treatment time compared to other impregnation methods; besides, it offers a larger surface area, microporosity and yield of the obtained activated carbon (AC) [25]. In chemical impregnation, the carbonized char material is impregnated with an oxidizing agent for dehydration by mixing or kneading with a concentrated solution of acid or base. The impregnation procedure increases the porosity and surface area and widens the existing micropores and mesopores of the char matrix [26]. The internal surface area of the AC can reach values up to 500 to 1500 m^2^/g [26]. The common chemical reagents applied at the industrial scale for the impregnation of biochar include ZnCl_2_, H_3_PO_4_, NaOH, KOH, and K_2_CO_3_ [24,27].

It is worth mentioning that the properties of the produced AC strongly depend on the type of precursor biomass and the operating conditions during pyrolysis (Ahmad et al., 2019). The lignin and cellulose content of the plant texture greatly affects the microstructure of the produced AC. Previous studies have shown that, the higher the lignin content in the biomass, the higher the biochar yield and micropores surface area [24]. In addition, the operating conditions, including temperature and pyrolysis residence time, play an important role in the yield and quality of the biochar produced. Generally, when conducting the pyrolysis at low temperatures (~500^o^C), high residence time is favorable for producing high-quality biochar [28]. Most of the previous adsorption studies in the literature have mainly focused on wastewater treatment; however, biogas purification using biochar adsorbents was lightly investigated.

Concerning the novelty of this work, in Palestine, the biogas production is quite limited, with only one full scale industrial-scale biogas plant that is in operation. In this plant, cow manure is being used as a feedstock to the biodigester. The generated biogas is purified from H_2_S using an expensive commercial AC filter. This study responds to a real need of the biogas industry in the country to provide a relatively cheap biogas purification technology that can also be applied in other developing countries in Asia, Africa and South America. Thus, it is hypothesized that the expensive AC can be replaced with different low-cost and effective ACs that can be produced from widely available biomass wastes such as spent coffee grains, eucalyptus barks and almond shells. The objective of the present study was to prepare low-cost biochar adsorbents from locally available biomass wastes for application in the purification of biogas from H_2_S using packed-bed adsorption system. The effect of the type of precursor biomass waste, impregnation agent and the packed-bed characteristics on the H_2_S removal efficiency was tested in this study.

## 2. Experimental: materials and methods

### 2.1. Biomass precursors and biogas composition

Coffee grains (COF) waste was collected from a local home kitchen (Hebron, Palestine). Eucalyptus barks (EUC) and almond shells (ALM) were collected from a local home garden (Hebron, Palestine). Concerning impregnation agents, potassium hydroxide (KOH) and zinc chloride (ZnCl_2_) (90%, Albemarle, Louisiana, Florida, USA) were used in this study. Biogas was obtained from the Al-Jebrini biogas facility, Al-Dhaheria, Hebron, Palestine. The composition of the biogas stream was as follows: 67.30% CH_4_, 31.90% CO_2_, 0.20% O_2_, 0.07% H_2_S, 0.53% other minor gases. The biodigester was fed with biomass waste obtained from Al-Jebrini cow farm Al-Dhaheria, Hebron. It consists of 20 ton/day of solid cow manure and 60 ton/day of liquid manure mixture (i.e. the solid cow manure mixed with wastewater from the milking station).

### 2.2. Pretreatment and pyrolysis of biomass wastes

The collected biomass wastes were washed with tap water to remove the impurities and dried in an oven (Daihan LabTech Co., Ltd., South Korea), at 105°C for 24 h. The samples were, then, smashed and sieved for 15 min using an auto sieve analysis shaker (Matest, Italy) with meshes # (10, 18, 40, 60 and 140). Particles retained on mesh #140 were used for subsequent analysis. The collected particles were filled in a tightly closed porcelain crucible, in order to isolate the oxygen and water vapor, and pyrolyzed at 500°C for 1 h inside a muffle furnace (labTech International Ltd. East Sussex, UK). The yield of the pyrolyzed materials was defined as the ratio of the weight of the produced biochar to the original biomass weight. Most of the samples were tested in triplicate for yield measurements and the average values are presented in this study with their standard deviation values (i.e. the SD, ± values).

### 2.3. Impregnation

For the impregnation of the samples, distilled water was added to the pyrolyzed biomass at a ratio of 10:1 (*v/w*) (*V_water_:w_biomass_*) and the impregnation reagent was then gradually added into the mixture, at a ratio of 1:4 (*wt _reagent_: wt _biomass_*). The mixture was carefully stirred at 300 rpm and heated to a temperature of 80°C using a hotplate magnetic stirrer (labTech International Ltd. East Sussex, UK) until most of the liquid was evaporated. The samples were then dried in an oven, at 105°C for 24 h.

### 2.4. Adsorption studies in a packed-bed reactor and flow characteristics studies

**Figure 1.**
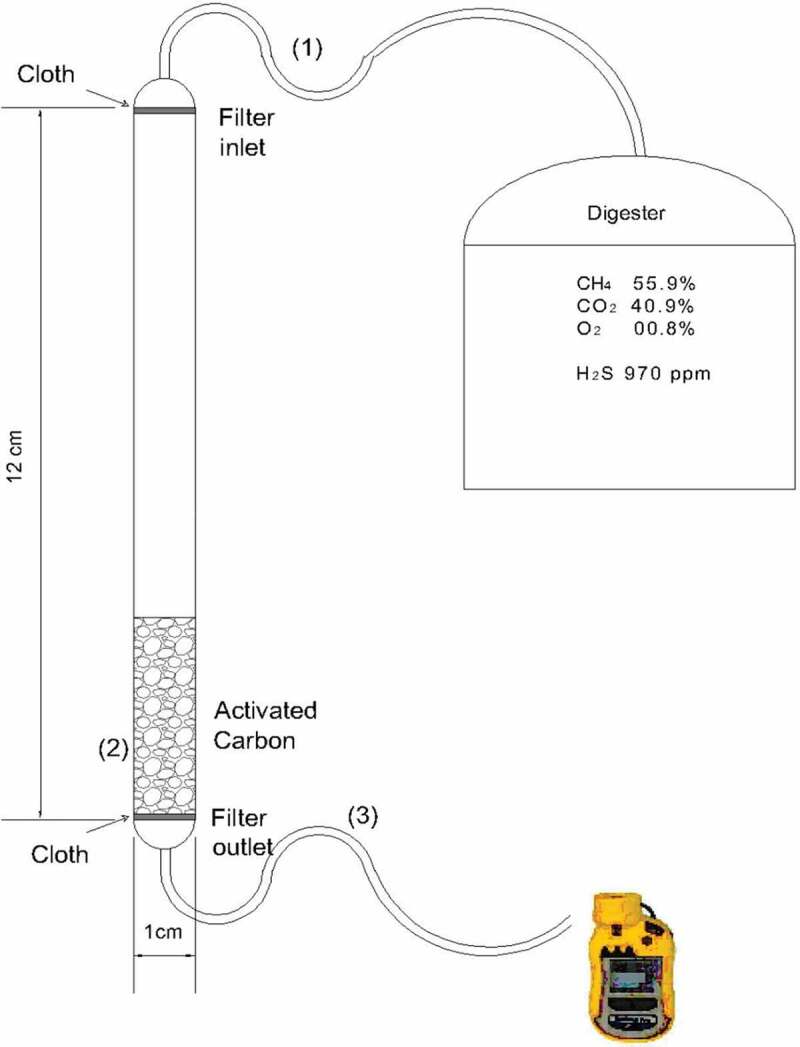
Experimental setup used for H_2_S adsorption test (1,3 – inlet and outlet connections, 2 – packed bed).

Adsorption experiments were performed using a packed-bed plastic filter with an internal diameter of 1 cm and a height of 12 cm (Figure 1). Two thin layers of a porous cloth were placed at the inlet and outlet of the filter in order to hold the AC particles. The bed was firstly run empty (without AC) in order to test the effect of the bed material and closing the cloth on the H_2_S adsorption behavior. The AC particles were then packed inside the bed at different heights of 2, 4, 6 and 8 cm, respectively. The biogas stream with an average H_2_S concentration of 970 ppm entered from the top of the filter bed and it was passed through the AC particles before leaving from the bottom of the packed bed reactor. The inlet and outlet concentrations of H_2_S were recorded during different time intervals. The removal efficiency of H_2_S was studied in terms of time course profiles, i.e. by sampling both the inlet and the outlet H_2_S concentrations. Thus, the breakthrough curves of the H_2_S adsorption inside the packed-bed were established by plotting the ratio of the outlet concentration to the initial concentration ratio (c/c_o_) as a function of the operational time.

The biogas flow rate was measured using the water displacement method [29] and was kept constant at approximately 1.5–1.7 L/min. The flow through the bed was characterized by estimating the Reynold’s number of the flow, as given in Equation (1) [30]. The bed characteristics and operating conditions are listed in Tables 1 and 2, respectively.

(1) $$Re{\;}_{p}=\frac{\rho \;U\;D}{\left(1-\varepsilon \right)\mu }$$

**Table 1.** The packed bed, biogas characteristics, and operating conditions.

Variable	Symbol	Value	Unit
Density (biogas)	ρ	1.17	kg/m^3^
Dynamic viscosity (biogas)	µ	1.26 × 10^–5^	Pa.s
Flow rate	Q	1.5	L/min
Particles average diameter	x	177 × 10^–6^	m
Bed cross-sectional area	A	7.85 × 10^–5^	m^2^
Superficial velocity	U	0.318	m/s

**Table 2.** Void fraction and pressure drop for the adsorption bed packed with various types of biochar.

Biochar	*є*	Re_p_	ΔP/H (kpa/m)	ΔP (kpa)
EUC	0.7	984.28	7.01	0.14
ALM	0.63	798.07	14.23	0.28
COF	0.67	894.81	9.54	0.19

where *ρ* is the gas density (Kg/m^3^), *U* is the superficial fluid velocity through the bed (m/s), *D* is the bed diameter (m), *μ* is the fluid dynamic viscosity (pa.s), and *є* is the void fraction of the bed (dimensionless). The values of *ε* were determined using Equation (2):

(2) $$\varepsilon =\frac{V_{V}}{V_{T}}$$

where *V_V_* is the void volume, which was measured using the fluid saturation method [31], and *V_T_* is total volume of the packed-bed.

The pressure drop for the flow through the bed was calculated using the Carman–Kozeny equation [30], as follows:

(3) $$\frac{\Delta p}{H}=180\;\frac{\mu U}{x^{2}}\frac{{\left(1-\varepsilon \right)}^{2}}{\varepsilon^{3}}+1.75\;\frac{\rho U^{2}}{x}\frac{(1-\varepsilon )}{\varepsilon^{3}}$$

where *Δp* is the pressure drop (pa), *H* is the height of the bed (m), and *x* is the particle diameter (m).

### 2.5. Adsorption capacity

**Figure 2.**
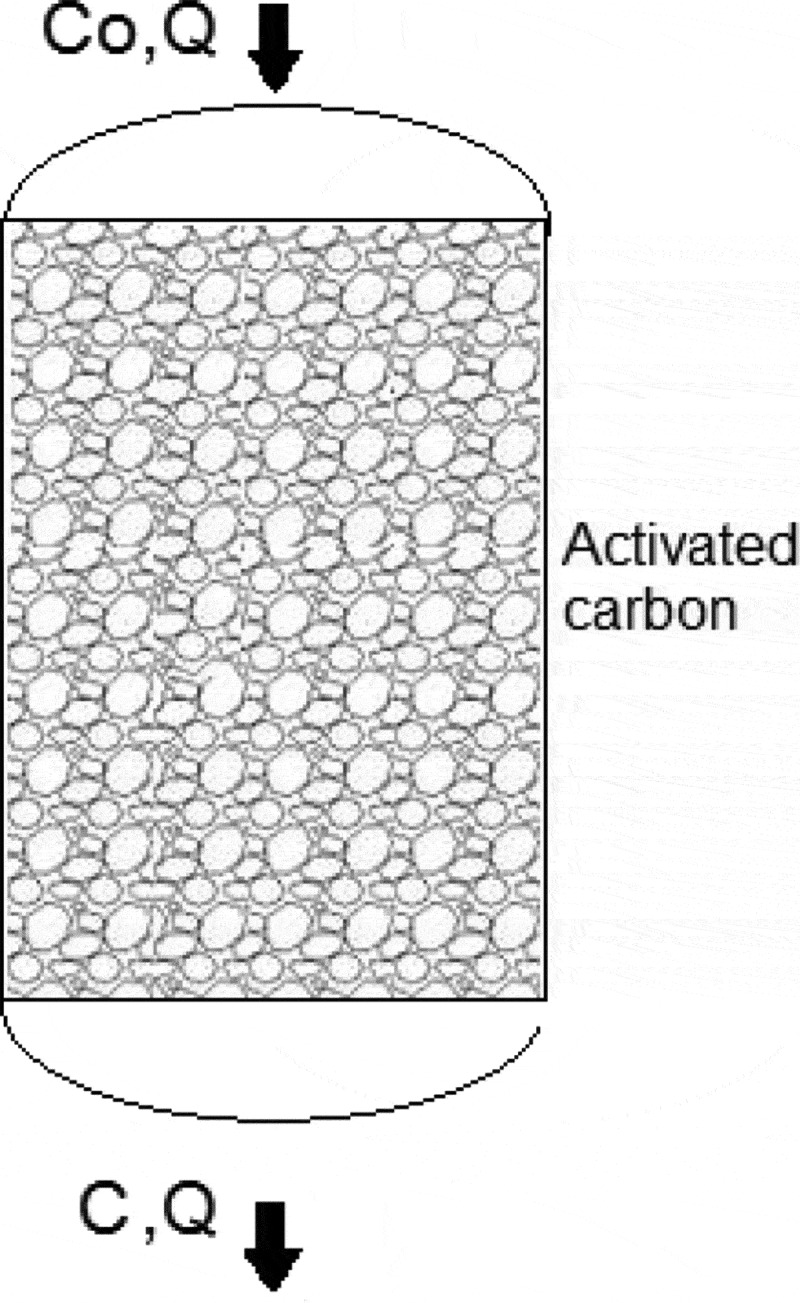
Activated carbon-based packed bed (*C* and *Q* are the H_2_S concentration and flow rate, respectively).

**Figure 3.**
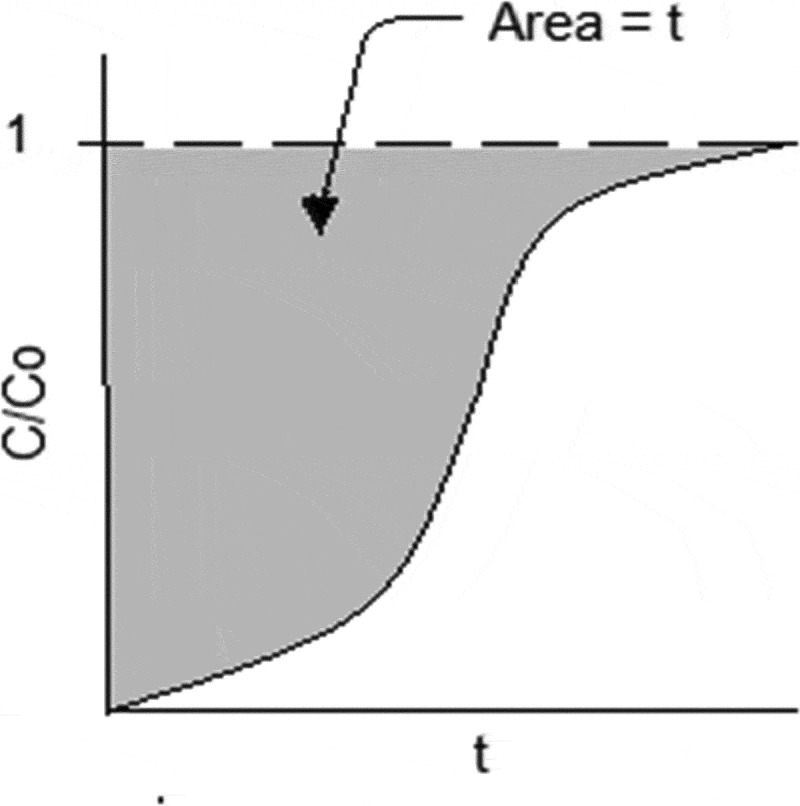
Breakthrough curve achieved after H_2_S adsorption.

The total amount of H_2_S adsorbed onto the AC particles was determined by applying mass balance principles on the packed-bed, as shown in Figure 2. The general mass balance equation given in Equation (4) was applied.

(4) $$\begin{array}{rl} & Total\;H_{2}\;Saccumulation\\ & \;\;\;\;\;\;\;\left(i.e.\;total\;amount\;of\;adsorbed\;H_{2}S\right)\\ & \;\;\;\;\;\;\;=input-output \end{array}$$

(5) $$Rate\;of\;accumulation\left(R\right)=\left(c_{o}\times Q\right)-\left(c\times Q\right)$$

where *c_o_* is the inlet concentration of H_2_S (g/L), *c* is the outlet concentration of H_2_S (g/L) and *Q* is the flow rate (m^3^/min).

(6) $$\int_{0}^{\infty }Rdt=\;\int_{0}^{\infty }(c_{o}\times Q-c\times Q)dt$$

(7) $$\int_{0}^{\infty }Rdt=Qc_{o}\int_{0}^{\infty }(\frac{c_{o}-c}{c_{o}})dt$$

(8) $$Total\;accumulation\;=\;\int_{\infty }^{0}(1-\frac{c}{c_{o}})dt$$

where *t* is the adsorption time (min). The integral $\overset{\infty }{\underset{0}{\int }}(1-\frac{c}{c_{o}})dt$ in Equation (8) can be obtained from the shaded area of the breakthrough curve as shown in Figure 3.

The adsorption capacity of AC can be obtained from Equation (9) as follows:

(9) $$q=\frac{m_{H2S}}{m_{AC}}$$

where *q* is the adsorption capacity of AC (mg H_2_S/g AC), *m_H2S_* is the total amount of H_2_S (mg) accumulated as given in Equation (8) and *m_AC_* is the mass of AC (g) filled in the adsorption bed.

## 3. Results and discussion

### 3.1. Biochar yield

The yield of the produced biochar from the pyrolysis process was determined for the three types of biomass waste and the results are shown in Table 3. The highest yield of 36% *w/w* was obtained with EUC followed by ALM (28.5%) and COF (24%), respectively. The difference in the yield of the biochar might be ascribed to the difference in the lignin and cellulose content; the higher the lignin content, the higher the yield [28]. Therefore, the EUC showed the highest yield due to the fact that its lignin content is the highest among others (Table 3) [7,32,33].

**Table 3.** The yield, H_2_S adsorption capacity, and cellulose and lignin contents of various biochar types.

Biomass	Yield (%)	Adsorption capacity (mg H_2_S/g AC)	Lignin content (% *w/w*)	Cellulose content (% *w/w*)	Reference
EUC	36 (±3.13)	490	26–33	55–68	[32]
ALM	28.5 (±0.71)	230	24.8	58	[7]
COF	23.87 (±1.96)	22	23.9	51	[33]

### 3.2. Effect of biomass precursor type on the adsorption characteristics

**Figure 4.**
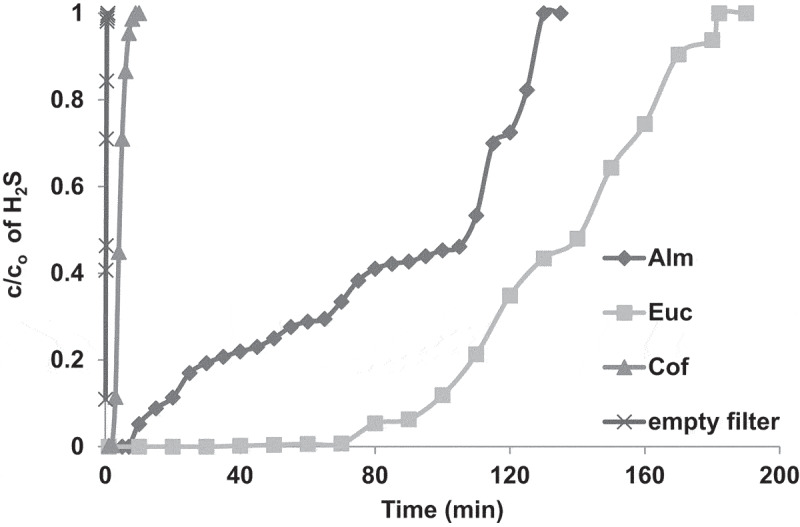
Breakthrough curves of H_2_S adsorption in packed bed of EUC, ALM, COF activated with KOH and empty filter. The experiments were performed at ambient air temperature, gas flow rate (1.5 L/min), bed height (2 cm) and H_2_S inlet concentration (970 ppm).

The effect of the type of biomass waste on the adsorption of H_2_S was investigated. Figure 4 shows the breakthrough curves of H_2_S adsorption with an empty bed and when the bed was packed with 2 cm of EUC, ALM and COF biochar activated with KOH, respectively. As seen from the results, when the bed was empty, the breakthrough curve was very steep and the outlet concentration rapidly increased to reach its inlet concentration within about 45 s; indicating that the amount H_2_S adsorbed onto the cloth and bed material is almost negligible. On the other hand, when the bed was charged with the AC material, the breakthrough time dramatically increased compared with those observed when the bed was empty and was highly dependent on the type of biomass precursor material used. At the beginning of the adsorption process, the outlet H_2_S concentration dramatically dropped to less than 10 ppm for the three samples; indicating high adsorption (H_2_S removal) efficiencies. However, with operational time, the outlet H_2_S concentration increased at different rates depending on the AC sample tested in this study. For instance, with COF, the breakthrough curve was steeper than EUC and ALM. The outlet concentration remained around zero (i.e. 100% removal efficiency) for about 3 min and the H_2_S outlet concentration rapidly increased to reach its inlet concentration after only 10 min. The ALM performed better than the COF; as the outlet concentration remained around zero for about 7 min; thereafter, it gradually increased to reach to its inlet concentration after 130 min (Figure 4).

The highest removal efficiency was observed with EUC as the outlet concentration remained almost zero, with a removal efficiency of about 100% for 45 min. However, after that, it slowly increased to reach its initial value after 180 min. The adsorption capacity of the AC to the H_2_S, as defined in Equation (9), was calculated for the three types of biomass precursors as shown in Table 3. As seen from the results, the high adsorption capacity observed in this study indicated better adsorption performance. The EUC showed the highest adsorption capacity of about 690 (mg H_2_S/g AC) followed by ALM and COF, respectively. The difference in the adsorption efficiencies and capacities of H_2_S with respect to the type of AC might be explained as follows: The porosity and microstructure of AC are usually strongly affected by the original plant texture, i.e. its lignin and cellulose contents. The lignin content is responsible for the formation of macropores of AC, whereas the cellulose content is mainly responsible for micropores formation [28]. The porosity of the AC obtained from COF (both types micro- and macro-porous) is, therefore, expected to be lower than EUC and ALM, due to the fact that it has a lower content of cellulose and lignin than EUC and ALM, respectively. This explains the lower adsorption capacity of the COF compared with the other ACs tested in this study. On the other hand, the EUC has the highest cellulose and lignin contents (Table 3) and thereby, one would assume its porosity to be the highest among others, which consequently, rendered the highest adsorption efficiency and capacity. Similar effects of the type of biomass precursor on the H_2_S adsorption capacity were observed in other studies using different types of biochar [34,35]

### 3.3. Effect of impregnation reagent on the adsorption characteristics

**Figure 5.**
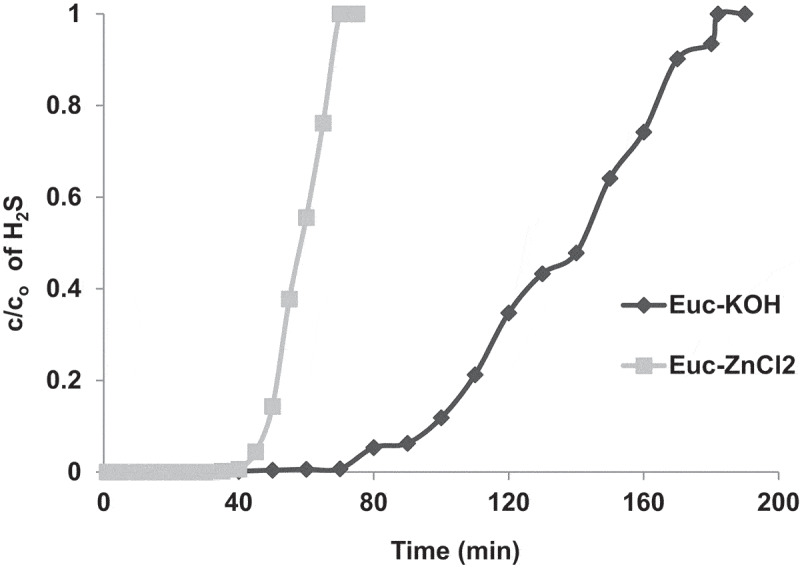
Breakthrough curves of H_2_S adsorption in bed packed of EUC-KOH and EUC-ZnCl_2_. The experiments were performed at ambient air temperature, gas flow rate – 1.5 L/min, bed height – 2 cm and inlet H_2_S concentration of 970 ppm.

Concerning the effect of the type of biomass, the influence of the impregnation reagent on the adsorption efficiency and capacity was investigated. Figure 5 shows the adsorption breakthrough curves and removal efficiencies for EUC impregnated with KOH and ZnCl_2_, respectively. One could clearly see that the adsorption efficiency of EUC is highly affected by the impregnation reagent. The adsorption performance of EUC impregnated with KOH was much better than with ZnCl_2_. For instance, the breakthrough time with KOH was higher than ZnCl_2_; it took about 180 min for the H_2_S outlet concentration to reach its initial value with KOH compared with only 70 min in the case of ZnCl_2_ (Figure 5). Furthermore, the KOH rendered higher adsorption capacity compared with ZnCl_2_.

The effect of the impregnation reagent on the breakthrough capacity and adsorption efficiencies can be ascribed to the difference in chemical affinity between the properties of H_2_S in the gas phase and the impregnation reagent. The adsorption of H_2_S is usually governed by local pH within the pores of the AC, as suggested in the literature [36]. An alkaline pore media, as in the case of KOH, promotes the formation of elemental sulfur through the reaction with the OH group [37], Equation (10), which increases the H_2_S removal efficiency. On the other hand, the acidic media, as in the case of ZnCl_2_, limits the dissociation of H_2_S; which consequently limits its oxidation to elemental sulfur and thereby reduces the overall H_2_S removal [36,38]. In addition, the impregnation reagent also affects the porosity of the AC. Previous studies have indicated that ZnCl_2_ creates mesoporous structure within the AC, whereas KHO is favorable for creating microporous AC which is generally better for adsorption [39].

(10) $$H_{2}S+2OH^{-}\to S^{2-}+2H_{2}O$$

### 3.4. Effect of bed height on the adsorption characteristics

**Figure 6.**
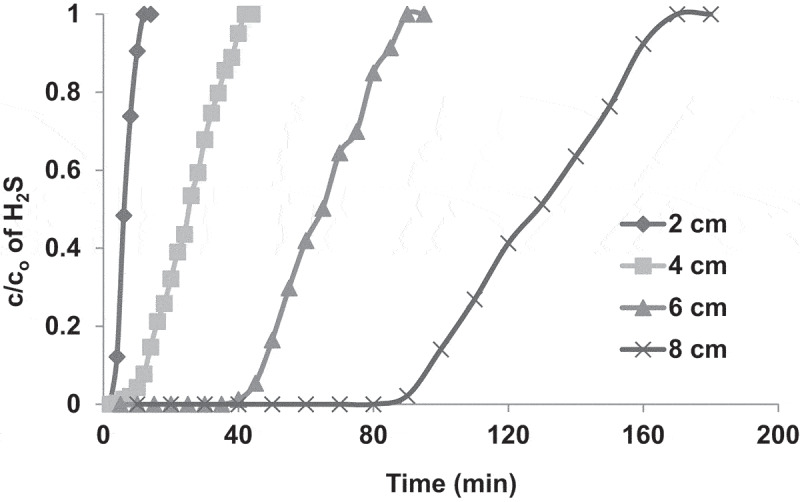
Adsorption breakthrough curves of H_2_S adsorption in packed bed of COF activated with KOH. The experiments were performed at ambient air temperature, gas flow rate of 1.7 L/min, pressure drop of 9.36 kpa/m, H_2_S inlet concentration (930 ppm) and bed heights of 2, 4, 6 and 8 cm.

**Figure 7.**
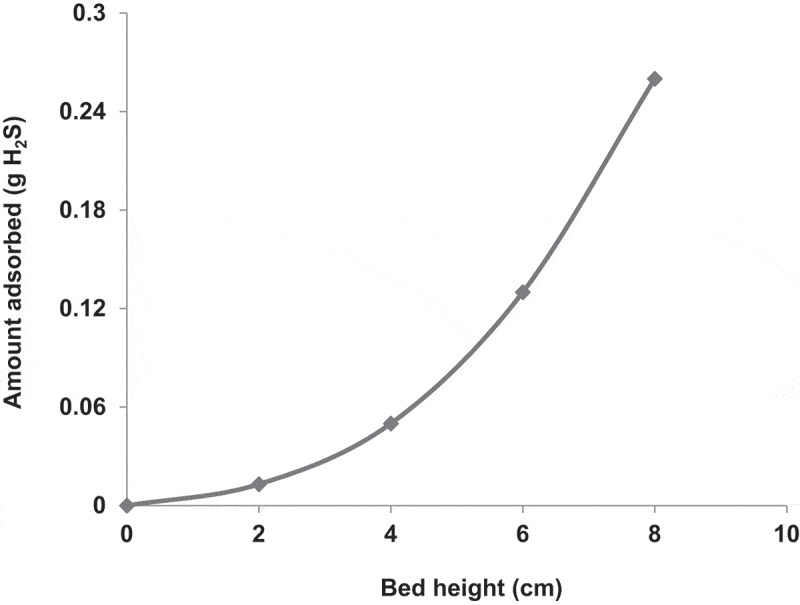
Bed height versus the amount of H_2_S adsorbed in the packed bed.

To study the effect of the bed height, the adsorption bed was charged with COF AC at different heights of 2, 4, 6 and 8 cm, respectively. The obtained adsorption breakthrough curves are shown in Figure 6. The results indicated that the higher the bed height, the higher the adsorption efficiency and the longer the time required for the saturation of the AC. For instance, at a bed height of 2 cm, the c/c_o_ ratio increased rapidly from 0 to 1 within about 10 min and this time gradually increased with increasing the bed height to reach about 170 min at a bed height of 8 cm. Increasing the bed height means charging the bed with larger amount of AC and consequently increasing the total surface area and the total number of adsorption active sites. Furthermore, a longer AC bed provides a longer contact time between the adsorbent and the adsorbate, which increases the the amount of adsorbed H_2_S, as shown in Figure 7.

Future studies should focus on the following aspects in order to upgrade lab-scale studies to pilot and semi-industrial scale experiments: (i) to determine the performance of adsorption columns under the influence of a mixture of pollutants that represents the typical composition of biogas (i.e. H_2_S, CO_2_, water vapor, siloxane, O_2_, CH_4_ and impurities such as siloxane), (ii) understand the mechanism of adsorption such as electrostatic attraction, interaction between the pollutant and the active sites, and partition in non-carbonized portion of the adsorbents, (iii) perform long-term adsorption studies in continuous systems and identify the deterioration of adsorption capacity of adsorbents under the influence of changing moisture contents of the biogas.

### 3.5. Mechanism of H_2_S removal

In nature, bacterial species could convert the different species of sulfur into elemental sulfur after complete oxidation. However, this also depends on the different phases of the sulfur cycle, and the environmental conditions such as pH, temperature, the presence of other competing ions and also on the aerobic, anoxic or anaerobic zones. However, in the case of adsorption-based mechanisms, the pattern of H_2_S removal might change depending on the present of sulfur compounds in the gas or liquid phase. Xu et al. [40] studied the H_2_S sorption capacities of pig manure and sewage sludge derived biochar for both static and dynamic systems, and revealed that the partial oxidation of H_2_S into elemental sulfur (S^0^) takes place in the biochar pores, while complete oxidation of H_2_S into sulfate ion (SO_4_^2-^) occurs on the surface of biochar. The authors also concluded that high moisture content in biochar would accelerate the complete oxidation of H_2_S, strong alkalinity of biochar helps to achieve high H_2_S sorption ability, and mineral-rich biochar performs key roles in the conversion of final sulfur compound. Xu et al. [40] proposed the following H_2_S removal mechanism (Equations (11)–(15)) by pig manure and sewage sludge derived biochar:

(11) $$H_{2}S_{\left(gas\;phase\right)}\to \;H_{2}S_{\left(adsorbed\;phase\right)}\to \;H_{2}S_{\left(liquid\;phase\right)}$$

(12) $$H_{2}S_{\left(liquid\;phase\right)}+OH^{-}\to H{S^{-}}_{\left(adsorbed\;phase\right)}+H_{2}O$$

(13) $$H{S^{-}}_{\left(adsorbed\;phase\right)}+O_{2}\to \;S^{0}$$

(14) $$H{S^{-}}_{\left(adsorbed\;phase\right)}+O_{2}+H_{2}O\to S{O_{4}}^{2-}\left(major\;in\;pig\;manure\;biochar\right)$$

(15) $$S{O_{4}}^{2-}+Ca^{2+}+2H_{2}O\to CaSO_{4}2H_{2}O\left(major\;in\;sewage\;sludge\;biochar\right)$$

### 3.6. Performance of different adsorbents and practical applications

Based on the literature review, woodchips derived biochar were found to be efficient for the removal of H_2_S (removal efficiency > 98%) at initial H_2_S concentration of 105, 510 and 1020 ppm_v_, pH 8.0, and moisture content in the range of 80–85% [41]. Agricultural or forest waste such as rice hull, bamboo, camphor tree can be used as a valuable source to produce biochar because of their high H_2_S adsorption capacity. However, the adsorption capacity depends on pyrolysis temperature and pH, for example, breakthrough time of an adsorbent increases with the increase of pyrolysis temperature [34]. According to Fellah [42], different zeolite-based adsorbents can also be used for the removal of gas phase H_2_S. The important features of this adsorbent can be stated as follows: (i), high surface area, (ii) very high adsorption capacity, (iii) good selectivity, (iv) regenerability and (v) good resistance to high temperatures that are usually expected in industrial emissions. In another recent study, Hassankiadeh and Hallajisani [43], tested the performance of Molybdenum oxide nano-particles for H_2_S removal wherein the parameters such as temperature (65–89°C), pressure (10–19 bar), initial concentration of H_2_S in the gas phase (38–73 ppm), and space velocity (0.018–0.045 m s^−1^) were varied. The results from that study showed a maximum adsorption capacity of 0.081 and 0.074 g H_2_S/g Molybdenum oxide using non-spherical and spherical Molybdenum oxide sorbent, respectively. In another study involving packed bed systems, Coenen et al. [44] studied the adsorption behavior of H_2_S and the influence of steam using commercially available potassium-promoted hydrotalcite. Based on the results obtained from packed bed systems, the authors showed that the operating temperature during adsorption has significant effect on the adsorption behavior. At high and low temperatures (i.e. 500°C and 300°C), different adsorption mechanisms namely chemisorption mechanism with high activation energy and physical adsorption were reported. It has also been reported that the carbonization temperatures could significantly affect the surface properties of an adsorbent. Wang et al. [45], modified the properties of activated carbon samples using a carbonization temperature of 450°C and KOH activation at 750 °C. For H_2_S removal, the authors reported a maximum adsorption capacity of ~98.0 mg/g, and the Langmuir isotherm model was found to describe the mechanism of adsorption of H_2_S.

From a practical application viewpoint, the efficient operation of a biofilter depends on the selection of a proper packing material [46] as well as the characteristics (e.g. porosity, water retention capability, capacity to hold microbial community) of the selected support media [47]. Compost biofilter is considered as one of the key biological processes to treat waste gases and to control odor. However, the performance of any compost-based biofilter depends on factors such as the type of filter media, gas flow rate, solubility of the pollutants, its concentration and biodegradability of the substrate [48–50]. As there is very limited information in the literature about effectiveness of biochar-based biofilter for H_2_S and volatile organic compounds (VOC) removal, future studies should focus on designing reactors using packing materials such as Eucalyptus, almond shells and coffee grains and evaluate the performance of these reactors in terms of the following parameters: (i) elimination capacity, (ii) critical inlet loading rate, (iii) maximum tolerable pollutant concentrations, (iv) capacity to handle transient or shock loads of H_2_S and VOCs and (v) perform intelligent control operation of the reactor using artificial intelligence techniques.

## 4. Conclusions

The results of this study confirm that AC could be successfully synthesized from various types of locally available biomass wastes and they are technically effective in purifying biogas from H_2_S. The yield of carbonization was dependent on the type of biomass; i.e. EUC rendered the highest yield of 35% followed by ALM (28%) and COF (23%), respectively. The type of biomass precursor, impregnation reagent and bed height strongly affected the removal efficiency of H_2_S. The highest adsorption capacity and removal efficiencies were obtained with EUC followed by ALM and COF, respectively. As an impregnation reagent, KOH gave the higher adsorption efficiency and capacity than ZnCl_2_. Increasing the bed height increases the adsorption capacity and H_2_S removal efficiency. Based on the obtained results, for Palestine and other developing countries, it is recommended to use EUC biochar impregnated with KOH for achieving high H_2_S removal efficiency from the biogas.

## Research highlights

- Hydrogen sulfide (H_2_S) removal by adsorption from biogas was tested in this study
- Effect of the type of precursors, impregnation reagent and bed height was studied
- KOH activated packing material showed good adsorption efficiency and capacity
- Increasing the bed height increased the H_2_S removal efficiency in the packed bed

## References

[1] Liu S, Yang G, Fu J, et al. Synchronously enhancing biogas production, sludge reduction, biogas desulfurization, and digestate treatment in sludge anaerobic digestion by adding K_2_FeO_4_. Environ Sci Pollut Res. 2018;25(35):35154–35163. 10.1007/s11356-018-3438-4 30328043

[2] Rahman KM, Harder M, Woodard R. Energy yield potentials from the anaerobic digestion of common animal manure in Bangladesh. Energy Environ. 2018;29(8):1338–1353.

[3] Rusmanis D, O’Shea R, Wall DM, et al. Biological hydrogen methanation systems – an overview of design and efficiency. Bioengineered. 2019;10(1):604–634. 31679461 10.1080/21655979.2019.1684607 PMC6844437

[4] Koyande AK, Show P-L, Guo R, et al. Bio-processing of algal bio-refinery: a review on current advances and future perspectives. Bioengineered. 2019;10(1):574–592. 31668124 10.1080/21655979.2019.1679697 PMC6844430

[5] Wainaina S, Kumar Awasthi, Taherzadeh MJ. Bioengineering of anaerobic digestion for volatile fatty acids, hydrogen or methane production: A critical review. Bioengineered. 2019;10(1):437–458. 31570035 10.1080/21655979.2019.1673937 PMC6802927

[6] Verma S, Das LM, Kaushik SC. Effects of varying composition of biogas on performance and emission characteristics of compression ignition engine using exergy analysis. Energy Convers Manag. 2017;138:346–359.

[7] Nabais JMV, Laginhas CEC, Carrott PJM, et al. Production of activated carbons from almond shell. Fuel Process Technol. 2011;92(2):234–240.

[8] Yuan J, Du L, Li S, et al. Use of mature compost as filter media and the effect of packing depth on hydrogen sulfide removal from composting exhaust gases by biofiltration. Environ Sci Pollut Res. 2019;26(4):3762–3770. 10.1007/s11356-018-3795-z 30539397

[9] Awe OW, Zhao Y, Nzihou A, et al. A review of biogas utilisation, purification and upgrading technologies. Waste Biomass Valorization. 2017;8(2):267–283.

[10] Shen M, Zhang Y, Hu D, et al. A review on removal of siloxanes from biogas: with a special focus on volatile methylsiloxanes. Environ Sci Pollut Res. 2018;25(31)1–16. 10.1007/s11356-018-3000-4 30187417

[11] Tilahun E, Sahinkaya E, Çalli B. A hybrid membrane gas absorption and bio-oxidation process for the removal of hydrogen sulfide from biogas. Int Biodeterior Biodegrad. 2018;127:69–76.

[12] Khoshnevisan B, Tsapekos P, Alfaro N, et al. A review on prospects and challenges of biological H_2_S removal from biogas with focus on biotrickling filtration and microaerobic desulfurization. Biofuel Res J. 2017;4(4):741–750.

[13] Koparal A, Un UT, Ogutveren UB. Electrochemical oxidation of sulfite ions in aqueous solutions. Int J Environ Pollut. 2004;21(6):579–587.

[14] Biswas RA, Pandey RA, Chakrabarti T, et al. A chemo-biological treatment of scrubbing water from power plants with recovery of value-added products. Int J Environ Pollut. 2010;43(1/2/3):129–142.

[15] Peluso A, Gargiulo N, Aprea P, et al. Nanoporous materials as H_2_S adsorbents for biogas purification: a review. Sep Purif Rev. 2019;48(1):78–89.

[16] Binh HTT, Long NQ. Selective adsorption of H_2_S in biogas using zeolite prepared by microwave-assisted method. Energy. 2016;52:56.

[17] Machač P, Martinec M. High-temperature separation of H_2_S from producer gas produced of biomass gasification by artificially prepared sorbents. In J Environ Sci Technol. 2018;16(8):3971–3978.

[18] Piergrossi V, Fasolato C, Capitani F, et al. Application of Raman spectroscopy in chemical investigation of impregnated activated carbon spent in hydrogen sulfide removal process. Int J Environ Sci Technol. 2019;16(3):1227–1238.

[19] Wang S, Nam H, Nam H. Utilization of cocoa activated carbon for trimethylamine and hydrogen sulfide gas removals in a confined space and its techno‐economic analysis and life‐cycle analysis. Environ Prog Sustain Energy.2019;38(6):e13241

[20] Mohammadi A, Saadati Z, Joohari S. Comparison of the adsorption of H_2_S by ZnO-TiO_2_ and Ni-ZnO-TiO_2_ nanoparticles: an adsorption isotherm and thermodynamic study. Environ Prog Sustain Energy. 2019;38(6):e13258

[21] Ahmad A, Reddy SS. Performance evaluation of upflow anaerobic sludge blanket reactor using immobilized ZnO nanoparticle enhanced continuous biogas production. Energy Environ. 2019; 31(2):330–347.

[22] Sathya Priya D, Sureshkumar MV. Synthesis of *Borassus flabellifer* fruit husk activated carbon filter for phenol removal from wastewater. Int J Environ Sci Technol. 2019; 17(2):829–842

[23] Rodriguez Correa C, Hehr T, Voglhuber-Slavinsky A, et al. Pyrolysis vs. hydrothermal carbonization: understanding the effect of biomass structural components and inorganic compounds on the char properties. J Anal Appl Pyrolysis. 2019;140:137–147.

[24] Nor NM, Lau LC, Lee KT, et al. Synthesis of activated carbon from lignocellulosic biomass and its applications in air pollution control—a review. J Environ Chem Eng. 2013;1(4):658–666.

[25] Abdullah NS, Hussin MH, Sharifuddin SS, et al. Preparation and characterization of activated carbon from *Moringa Oleifera* seed pod. Cellulose. 2017;28:50.

[26] Ho N. Modeling hydrogen sulfide adsorption by activated carbon made from anaerobic digestion by-product. 2012;University of Toronto, Toronto, Canada, Master's Thesis.

[27] Ioannidou O, Zabaniotou A. Agricultural residues as precursors for activated carbon production—A review. Renew Sust Energ Rev. 2007;11(9):1966–2005.

[28] Tripathi M, Sahu JN, Ganesan P. Effect of process parameters on production of biochar from biomass waste through pyrolysis: A review. Renew Sust Energ Rev. 2016;55:467–481.

[29] Zhong W, Zhang Z, Luo Y, et al. Effect of biological pretreatments in enhancing corn straw biogas production. Bioresour Technol. 2011;102(24):11177–11182. 22000969 10.1016/j.biortech.2011.09.077

[30] Rhodes MJ, Rhodes M. Introduction to particle technology. West Sussex, England:John Wiley & Sons; 2008.

[31] Bear J. Dynamics of fluids in porous media. New York, NY:Courier Corporation; 2013.

[32] Dutt D, Tyagi C. Comparison of various eucalyptus species for their morphological, chemical, pulp and paper making characteristics. Indian J Chem Technol. 2011;18(2):145-151.

[33] Ballesteros LF, Teixeira JA, Mussatto SI. Chemical, functional, and structural properties of spent coffee grounds and coffee silverskin. Food Bioprocess Technol. 2014;7(12):3493–3503.

[34] Shang G, Li Q, Liu L, et al. Adsorption of hydrogen sulfide by biochars derived from pyrolysis of different agricultural/forestry wastes. J AirWaste Manage Assoc. 2016;66:8–16. 10.1080/10962247.2015.1094429 26447857

[35] Sethupathi S, Zhang M, Rajapaksha A, et al. Biochars as potential adsorbers of CH_4_, CO_2_ and H_2_S. Sustainability. 2017;9(1):121.

[36] Adib F, Bagreev A, Bandosz TJ. Effect of pH and surface chemistry on the mechanism of H_2_S removal by activated carbons. J Colloid Interface Sci. 1999;216(2):360–369. 10421743 10.1006/jcis.1999.6335

[37] Turk A, Sakalis E, Rago O, et al. Activated carbon systems for removal of light gases. Ann N Y Acad Sci. 1992;661(1 Frontiers of):221–228.

[38] Bandosz TJ. On the Adsorption/oxidation of hydrogen sulfide on activated carbons at ambient temperatures. J Colloid Interface Sci. 2002;246(1):1–20. 16290378 10.1006/jcis.2001.7952

[39] Okhovat A, Ahmadpour A. A comparative study of the effects of different chemical agents on the pore-size distributions of macadamia nutshell-based activated carbons using different models. Adsorpt Sci Technol. 2012;30(2):159–169.

[40] Xu X, Cao X, Zhao L, et al. Comparison of sewage sludge- and pig manure-derived biochars for hydrogen sulfide removal. Chemosphere. 2014;111:296–303. 24997932 10.1016/j.chemosphere.2014.04.014

[41] Kanjanarong J, Giri BS, Jaisi DP, et al. Removal of hydrogen sulfide generated during anaerobic treatment of sulfate-laden wastewater using biochar: evaluation of efficiency and mechanisms. Bioresour Technol. 2017;234:115–121. 28319759 10.1016/j.biortech.2017.03.009

[42] Fellah MF. Adsorption of hydrogen sulfide as initial step of H_2_S removal: A DFT study on metal exchanged ZSM-12 clusters. Fuel Process Technol. 2016;144:191–196.

[43] Hassankiadeh MN, Hallajisani A. Application of Molybdenum oxide nanoparticles in H_2_S removal from natural gas under different operational and geometrical conditions. J Pet Sci Eng. 2020;190:107131.

[44] Coenen K, Gallucci F, Hensen E, et al. Adsorption behavior and kinetics of H_2_S on a potassium-promoted hydrotalcite. Int J Hydrogen Energy. 2018;43(45):20758–20771.

[45] Wang S, Nam H, Nam H. Preparation of activated carbon from peanut shell with KOH activation and its application for H_2_S adsorption in confined space. J Environ Chem Eng. 2020;8(2):103683.

[46] Jaber MB, Couvert A, Amrane A, et al. Removal of hydrogen sulfide in air using cellular concrete waste: biotic and abiotic filtrations. Chem Eng J. 2017;319:268–278.

[47] Baltrėnaitė E, Baltrėnas P, Bhatnagar A, et al. A multicomponent approach to using waste-derived biochar in biofiltration: A case study based on dissimilar types of waste. Int Biodeterior Biodegrad. 2017;119:565–576.

[48] Morgan-Sagastume JM, Noyola A. Hydrogen sulfide removal by compost biofiltration: effect of mixing the filter media on operational factors. Bioresour Technol. 2006;97(13):1546–1553. 16051484 10.1016/j.biortech.2005.06.003

[49] Rene ER, Kar S, Krishnan J, et al. Start-up, performance and optimization of a compost biofilter treating gas-phase mixture of benzene and toluene. Bioresour Technol. 2015;190:529–535. 25827361 10.1016/j.biortech.2015.03.049

[50] Rene ER, Sergienko N, Goswami T, et al. Effects of concentration and gas flow rate on the removal of gas-phase toluene and xylene mixture in a compost biofilter. Bioresour Technol. 2018;248(Part B):28–35. 28844689 10.1016/j.biortech.2017.08.029

